# Plant Protein versus Dairy Proteins: A pH-Dependency Investigation on Their Structure and Functional Properties

**DOI:** 10.3390/foods12020368

**Published:** 2023-01-12

**Authors:** Qi Tang, Yrjö H. Roos, Song Miao

**Affiliations:** 1 Teagasc Food Research Centre, Moorepark, P61C996 Cork, Ireland; 2 School of Food and Nutritional Sciences, University College Cork, T12R229 Cork, Ireland; 3 China-Ireland International Cooperation Centre for Food Material Sciences and Structure Design, Fujian Agriculture and Forestry University, Fuzhou 350002, China

**Keywords:** plant protein, dairy protein, structural properties, functionality, pH-dependency

## Abstract

Plant proteins are constantly gaining attention as potential substitutes for dairy proteins, due to their suitable functionality and nutritional value. This study was designed to compare the structural and functional responses of different plant protein isolates (soy, pea, lentil, and chickpea) with two commonly used dairy protein (whey protein isolates and sodium caseinate) under different pH treatments (pH 3.0, 5.0, 7.0, and 9.0). The results showed that pH had a different alteration on the structural, surface properties and functional properties of plant and dairy proteins. Plant protein generally possessed a darker color, lower solubility, emulsifying properties, and foaming capacity, whereas their foaming stability and water holding capacity were higher than those of dairy proteins. Soy protein isolates were characterized by its comparable proportion of β-turn and random coils, zeta-potential, emulsifying (30.37 m^2^/g), and water-holding capacity (9.03 g/g) at alkaline conditions and chickpea protein isolates showed good oil-holding capacity (3.33 g/g at pH 9) among plant proteins. Further analysis confirmed that pH had a greater influence on the structural and functional properties of proteins as compared to protein sources, particularly at acidic conditions. Overall, this study might help processors select the appropriate plant protein as dairy alternatives for their target application in plant-based food products.

## 1. Introduction

Proteins, especially dairy proteins, are typically appealing ingredients for food applications since they can serve as gelling, emulsifying, or foaming agents. Whey protein (~20%) and casein (~80%) are the major protein components in bovine milk. The major protein components in whey protein isolates are α-lactalbumin (α-La), β-lactoglobulin (β-Lg), immunoglobulins (IgG), and bovine serum albumin (BSA). β-Lg, the dominate component (50–60%) of whey, is a small water-soluble protein with a compact three-dimensional globulin structure made up of nine distinct sheets folded into a globulon-like structure [1]. Sodium caseinate, obtained by adjusting pH of casein micelles to 4.6, washing and resolubilizing, is a commonly used dairy ingredient in the formulations of foods [2]. It contains four αs1-, αs2-, β-, and κ-casein fractions in a mass ratio of about 4:1:4:1.3 [3]. The tertiary structure of these casein proteins is poorly defined because they are rheomorphic [4].

It is nonetheless expected that in order to meet this increased global population demand (around 10 billion in 2050 and 11.2 billion in 2100), food production must increase by 60–70%, posing a major concern in food production [5]. The production and consumption of protein from livestock thus constitute the biggest environmental disturbances, affecting both carbon cycles and pollutant emissions, and resulting in land degradation and deforestation [6,7]. Therefore, finding reliable alternatives to dairy proteins is an important research priority due to climate change, world population growth, and biodiversity threats [8]. In recent years, plant proteins have become popular alternatives in sustainability-oriented applications owing to their inexpensive source, health benefits, soil fertility, and protein content (20–40%) [9]. Among emerging plant protein ingredients, soy, pea, lentil, and chickpea are mainly composed of 11S legumin (namely glycinin in soy) and 7S vicilin (namely β-conglycinin in soy) globulin-type storage proteins. The 11S globulins have a closely packed and rigid hexametric structure, while 7S globulins exist as a trimer [10].

Proteins derived from various sources usually have different structural and functional properties, and these properties can be severely affected by pH in very complex ways [11]. For example, β-Lg exists in solution as a spherical dimer at pH 5.5–7.5 at room temperature, however forms as octamers at pH between 3.5–5.1, whereas it dissociates into monomers at pH over 7.5 or below 3.0 [12,13]. 7S and 11S globulins can undergo a complex association–dissociation phenomenon in relation to their constituent polypeptides, subunits, and half-molecules under various pH conditions. The hexamer structure (11S) of glycinin also dissociates into the trimers (7S) on changing the pH from 7.6 (ionic strength = 0.5) to 3.8 (ionic strength = 0.03) [14]. Protein surface properties and related functional attributes can also be altered by adjusting the pH near or away from its isoelectric point [15]. Thus, their potential application will be greatly affected by these features since they will also affect food texture and organoleptic characteristics, which are crucial when making products, such as beverages, confectionary, dressings, and meat [16]. Several studies have examined the influence of pH on the functionality of certain proteins from plant or dairy sources. However, the difference in experimental conditions make it difficult to compare [17,18]. For example, it was reported that the charge of whey protein decreased from approximately +40 to 0 and −40 mV, while the associated emulsifying activity decreased from 102.5 to 41.5 and increased to 83.5 m^2^/g when pH increased from 3.0 to 5.0 and 7.0 [17]. However, a slight increase in emulsifying activity was reported when the pH of whey protein solutions was adjusted from 3.0 to 7.0 [19]. Therefore, it is important to systematically compare the structural and functional properties of plant and dairy proteins at different pH levels under precisely the same experiment conditions in order to assess the impact of pH and protein type on their functionality and potential application.

Thus, the present work reports the effect of pH (3, 5, 7, and 9) on the structural (fourier transform infrared spectroscopy), surface properties (surface charge, surface hydrophobicity, and intrinsic fluorescence) and functional properties (emulsifying, solubility, water/oil holding capacities, and foaming properties) of plant proteins (soy, pea, lentil, and chickpea) and dairy proteins (whey protein isolates and sodium caseinate). The findings of this study elucidated the influence of pH and protein type on functionality so that the functional properties could be tailored and utilized to improve the future application possibilities of plant proteins.

## 2. Materials and Methods

### 2.1. Materials

Pea (PPI), whey protein isolates (WPI), and sodium caseinate (SC) were purchased from Naturz Organics Europe (Helmond, The Netherlands), Carbery Group Limited (Cork, Ireland), and Sigma-Aldrich (St. Louis, MO, USA). Soy protein isolate (SPI) was kindly donated by Archer Daniels Midland Company (Decatur, IL, USA). All the commercial products were used without further purification. Lentil and chickpea protein flours were kindly provided by Fraunhofer (Munich, Germany) and Döhler GmbH (Darmstadt, Germany), respectively. Lentil (LPI) and chickpea protein isolates (CPI) were obtained by alkali extraction and acid precipitation, respectively [20]. All chemicals of analytical grade were used in this study.

### 2.2. Physicochemical Properties

#### 2.2.1. Chemical Composition

The content of total crude protein was determined by Kjeldahl method (crude protein using N × 6.25 for soy, pea, chickpea, and lentil proteins [21]; N × 6.38 for whey protein and sodium caseinate) [22]. The moisture and ash contents were analyzed by a LECO TGA-701 gravimetric oven (LECO Instruments, St. Joseph, MI, USA). The lipid content was determined by ORACLE + SMART 6 systems (CEM Corporation, Matthews, NC, USA). The carbohydrate content was calculated by difference.

#### 2.2.2. Color Properties

Chroma meter (CR-400, Konica Minolta Sensing, Inc., Osaka, Japan) was used to determine the color properties of plant and dairy protein isolates. L* (Lightness), a* (redness) and b* (yellowness) were used to describe the color values. Additionally, the whiteness index (preferred white and yellow) was calculated by Formula (1):

(1) $$Whiteness=100-\sqrt{{\left(100-L^{*}\right)}^{2}+a^{*2}+b^{*2\;}}$$

### 2.3. Structure Characteristics

#### 2.3.1. Sodium Dodecyl Sulfide-Polyacrylamide Gel Electrophoresis (SDS–PAGE)

SDS-PAGE of the protein isolates was performed to measure the molecular weight of protein fractions. The samples were diluted to 0.75 mg/mL in SDS solutions (1%, *w*/*v*) under nonreducing conditions overnight, and then centrifuged at 7000× *g* for 10 min. NuPAGE LDS sample buffer (7.5 μL, 4X, Invitrogen, Thermo Fisher Scientific, Cork, Ireland) and reducing agent (3 μL, 10X, Invitrogen, Thermo Fisher Scientific, Cork, Ireland) were added to the diluted protein samples (19.5 μL) before boiling at 70 °C for 10 min. Aliquots of 10 μL of protein and PageRuler Unstained Protein Ladder (10 μL) (Thermo Fisher Scientific Inc., Cork, Ireland) were loaded into 12% MINI-Protein^®^ TGX™ precast polyacrylamide gels (Bio-Rad Laboratories, Dublin, Ireland). Mini-PROTEAN Tetra System (Bio-Rad Laboratories, Dublin, Ireland) was performed at a voltage of 200 V for 30 min. Coomassie Blue R-250 was used to stain the gels for 2 h after electrophoresis, followed by an overnight destaining step. Epson Perfection V850 Pro scanner was used to scan the gels.

#### 2.3.2. Fourier Transform Infrared Spectroscopy Analysis (FT-IR)

Infrared spectra of the lyophilized protein samples were analyzed by FTIR spectroscopy (Bruker Tensor 27, Bruker Optik GmbH, Ettlingen, Germany) equipped with ATR cell (PIKE Technology Inc., Madison, WI, USA). The spectra were recorded from 900 to 4000 cm^−1^ with an average of 120 scans at a resolution of 4 cm^−1^. Baseline corrections and FTIR spectra deconvolutions and peak fitting of deconvolved FTIR spectra (amide I region, 1700–1600 cm^−1^) using the second-derivative Gaussian area method were conducted by Peakfit 4.12 software (Jandel Scientific Software, San Rafael, CA, USA) [19]. The area of each secondary structure was calculated as the percentage of each fitted area based on the integrated peak area.

### 2.4. Surface Characteristics

#### 2.4.1. Surface Charge

For zeta-potential (ζ) measurements, the protein solution of 1% (*w*/*v*) with different pH levels (from pH 2 to 11) were centrifuged at 8000× *g* for 20 min. The obtained supernatant was used for the measurement of zeta potential by a Zetasizer Nano ZS (Malvern Instruments Ltd., Worcestershire, UK) with a refractive index of 1.45 and 1.33 for proteins and water, respectively.

#### 2.4.2. Surface Hydrophobicity and Intrinsic Fluorescence

For surface hydrophobicity (Ho) measurement, 1-anilino-8-naphthalene sulfonate (ANS) was used as the fluorescent indicator. Several protein solutions were prepared with the concentration of 0.01 to 0.1% at different pH (3.0, 5.0, 7.0, 9.0). Protein solution (4 mL) was mixed with 20 μL of ANS solution (8.0 mM) for 10 s. These solutions were then incubated in the dark for 15 min, and the fluorescence intensity (FI) of these solutions were conducted by a Varian Cary Eclipse fluorescence spectrometer with the excitation and emission wavelengths of 365 and 484 nm, respectively. The protein surface hydrophobicity (Ho) was calculated as the slope of the plot of the corrected FI versus the protein concentration.

Fluorescence intensity (FI) was measured by a Varian Cary Eclipse fluorescence spectrometer at 290 and 420 nm for excitation and emission, respectively. The test was carried out on 0.5 mg/mL protein solutions at different pH 3.0, 5.0, 7.0, and 9.0.

### 2.5. Techno-Functional Properties

#### 2.5.1. Protein Solubility

Approximately 0.5 g protein samples were stirred with 20 mL deionized water overnight at ambient temperature, and then solutions were adjusted to pH 3.0, 5.0, 7.0, 9.0, 11.0. The solutions were mixed for 1h and then centrifuged at 10,000× *g* for 20 min at 25 °C. The protein content in the supernatant was determined by Kjeldahl method. The solubility (%) was calculated according to the following formula:

(2) $$Solubility\;\left(\%\right)=\frac{M_{1}}{M_{2}}\times 100\%$$

where *M*_1_ is the amount of protein in the supernatant and *M*_2_ is the amount of protein in protein sample.

#### 2.5.2. Emulsifying Properties

The protein solutions 1.0% (*w*/*v*) were prepared at different pH (3.0, 5.0, 7.0, and 9.0). The emulsions were then prepared by mixing protein solutions (9 mL) with soybean oil (3 mL) using an Ultra-Turrax at 20,000 rpm for 2 min. Freshly prepared emulsions (50 μL) were immediately taken out from the bottom of the bottle and mixed with 0.1% (*w*/*v*) sodium dodecyl sulfate solution. The initial absorbance (*A*_0_) and absorbance after 10 min (*A*_10_) of the diluted emulsion were measured at 500 nm using SpectraMax ABS Plus reader (Molecular Devices, San Jose, CA, USA). Emulsion activity index (EAI) and emulsion stability index (ESI) were calculated using Formulas (3) and (4), respectively.

(3) $$EAI\;\left(m^{2}/g\right)=2\times 2.303\times \frac{A_{0}\times N}{10,000\times \theta \times L\times C}$$

(4) $$ESI\;\left(\min\right)=\frac{A_{0}}{A_{0}-A_{10}}\times 10$$

where *C* is the concentration of the sample (0.01 g mL^−1^), *L* is the path length of the cuvette (1 cm), θ is the oil phase ratio (0.25), and *N* is the dilution factor (100).

#### 2.5.3. Foaming Properties

Foaming capacity (FC) and foaming stability (FS) of proteins were obtained by dispersing protein in distilled water and adjusting their pHs (3.0, 5.0, 7.0, and 9.0) using either 1.0 N NaOH or 1.0 N HCl to make the concentration of protein solutions 1.0% (*w*/*v*). Then, an Ultra-Turrax were used to mix 30 mL of protein suspension at 20,000 rpm for 2 min. Formulas (5) and (6) were used to calculate foaming capacity (FC) and foaming stability (FS), respectively [23].

(5) $$FC\;\left(\%\right)=\frac{\left(V_{1}-V_{0}\right)}{V_{1}}\times 100\%$$

(6) $$FS\;\left(\%\right)=\frac{V_{t}}{V_{1}}\times 100\%$$

where *V*_0_ and *V*_1_ denote the volumes of solution before and after mixing, and *V_t_* represents the volume of protein solution after 0.5, 1.0, 2, 2.0, 4.0 h storage, respectively.

#### 2.5.4. Water-Holding Capacity (WHC) and Oil-Holding Capacity (OHC)

The water-holding capacity (WHC) and oil-holding capacity (OHC) of protein samples were measured using the previous method with some changes [20]. Briefly, after mixing the protein powders with deionized water, the pH levels were adjusted (pH 3.0, 5.0, 7.0, and 9.0) and stirred for a further 40 min. The solutions were freeze-dried to obtain protein samples. One gram of the freeze-dried protein samples was mixed with 10 mL deionized water (sunflower oil for OHC) by a rotary mixer for 30 min and centrifuged for 20 min at 8000× *g*. After centrifugation, the supernatant was carefully decanted, and the remaining pellets were weighed. The WHC or OHC (g/g) were calculated as the Formula (7).

(7) $$WHC\;\left(\mathrm{or}\;OHC\right)\left(g/g\right)=\frac{M_{2}-M_{1}}{M_{2}}\;$$

where *M*_1_ is the weight of protein sample, *M*_2_ is the weight of wet or oily protein sample.

### 2.6. Hierarchical Clustering Analysis (HCA)

Hierarchical cluster analysis (HCA) was used to visualize and group the plant and dairy proteins under different pH treatment into clusters in terms of their similarity. It involves calculating the distance between individuals according to Euclidean distances to represent the dissimilarity performed by Origin software (Ver. 2019b, Origin Lab, Corporation, Northampton, MA, USA).

### 2.7. Statistical Analysis

All measurements were performed in triplicate, and results were expressed as mean ± standard deviation. Significantly differences (*p* < 0.05) in mean values were processed using Duncan’s mean comparison by SPSS 26.0 (SPSS Inc., Chicago, IL, USA). Origin software (Ver. 2019b, Origin Lab, Corporation, Northampton, MA, USA) was used to draw the graphs.

## 3. Results and Discussions

### 3.1. Physicochemical Characteristics

The proximate composition of the plant and dairy protein isolates are provided in Table 1. The protein content of all samples ranged from 78.66 (PPI) to 89.96% (SPI). For commercial proteins, all isolates exceeded the requirement of at least 80% protein content, except PPI (78.66%). Ash content, which reflects mineral concentration, was generally low (<5%), with highest value in SPI (4.74%) and lowest in WPI (2.72%) [24]. There was a relatively large difference in moisture content between protein isolates from different samples, with CPI (1.42%) being the lowest and PPI (7.59%) being the highest, which were comparable to previously published results [20]. All of the defatted samples had lower lipid contents (<5%), demonstrating the impact of the defatting process on the purity of protein.

**Figure 1:**
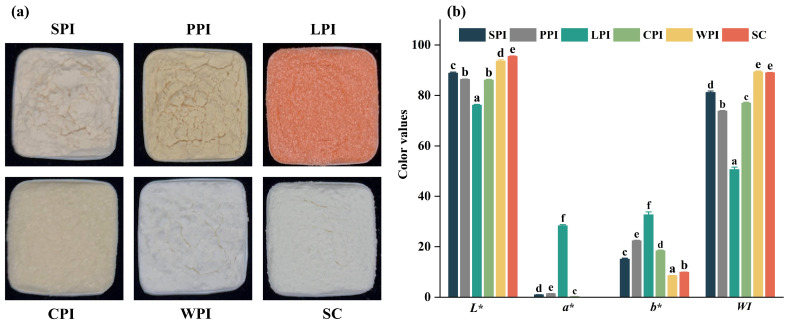
Visual appearance (**a**) and color values (**b**) of plant and dairy protein isolates. Different letters indicated significant different (*p* < 0.05) among protein samples under neutral conditions.

The color values showed that plant proteins showed brownish-black color (higher a* and b*), whereas dairy protein samples showed creamy-white color (higher L*) (Figure 1b). The variations in color properties are affected by various factors, such as the original color of the protein sources, protein purity, drying methods, and particle sizes [25]. LPI had significantly higher b* (*p* < 0.05) (32.68) and a* (28.30) values, and lower *WI* (50.63) and L* (76.17). Therefore, decolorization might be performed to improve the application of LPI to certain foods for color concern. The *WI* values of WPI (89.30) and SC (89.04) were significantly (*p* < 0.05) higher than SPI (81.22), CPI (76.93), PPI (73.79), and LPI (50.63), which corresponded to the visual appearance shown in Figure 1a.

### 3.2. Structural Analysis

#### 3.2.1. Protein Profile Analysis (SDS-PAGE)

**Figure 2:**
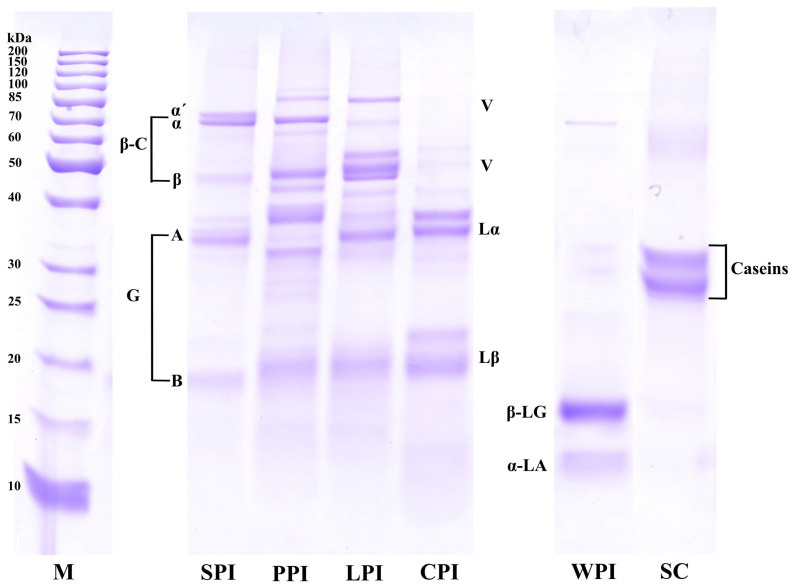
SDS-PAGE profiles of plant and dairy protein isolates (1% SDS). M: marker; SPI: soy protein isolates, PPI: pea protein isolates, LPI: lentil protein isolates, CPI: chickpea protein isolates, WPI, whey protein isolates, SC: sodium caseinate. G: glycinin (A: acidic subunit; B: basic subunit); β-C: β-conglycinin (α, α’, β-conglycinin polypeptides); L: legumin (α: acidic subunit; β: basic subunit); V: vicilin polypeptides; α-LA: α-lactalbumin; β-LG: β-lactoglobulin.

SDS-PAGE profiles of the plant and dairy protein isolates are shown in Figure 2. Globulins and albumins are the two major components in these plant proteins. 11S globulins and 7S globulins are chief components of globulins—named as glycinin and β-conglycinin in soy proteins and legumin and vicilin in PPI, LPI, and CPI, respectively. 11S globulins (320–400 kDa) is a hexamer consisting of different polypeptides in these plant protein isolates, and each subunit (~60 kDa) comprises a basic subunit (~20 kDa) and an acidic (~40 kDa) bonded by a disulfide bond [26]. 7S globulins (150–200 kDa) is a trimeric glycoprotein that mainly comprises several subunits associated via hydrogen bonding and hydrophobic rather than disulfide bonding. SPI samples showed the α’ (~79 kDa), α (~72 kDa), β (~47 kDa) subunits of 7S β-conglycincin fraction, and basic (~19 kDa) and acidic (~37 kDa) subunits of 11S glycinin fraction [27,28]. PPI samples showed the band of α (~40 kDa), β (~20 kDa), and α + β (~60 kDa) of 11S legumin subunits, α + β (~33 kDa) and α + β + γ (~50 kDa) of 7S vicilin, convicilin (~70 kDa), and lipoxygenase (~90 kDa) [29]. LPI samples exhibited bands of both 11S legumin protein subunits (α, ~40 kDa; β, ~20 kDa), 7S vicilin (~50 kDa), and lipoxygenase (~90 kDa) [29,30]. CPI also showed legumin α (~20 kDa), β (~40 kDa), and vicilin (~50 kDa) [31,32]. WPI samples shared similar bonds profiles with less intense α-La (~14 kDa) and more intense β-Lg (~18 kDa) [33]. The bands in the region of 25–35 kDa in the SC samples might be attributed to α_s1_, α_s2_, β and κ-casein, which are in line with the previous findings [34].

#### 3.2.2. Fourier Transform Infrared Spectroscopy (FT-IR) Analysis

**Figure 3:**
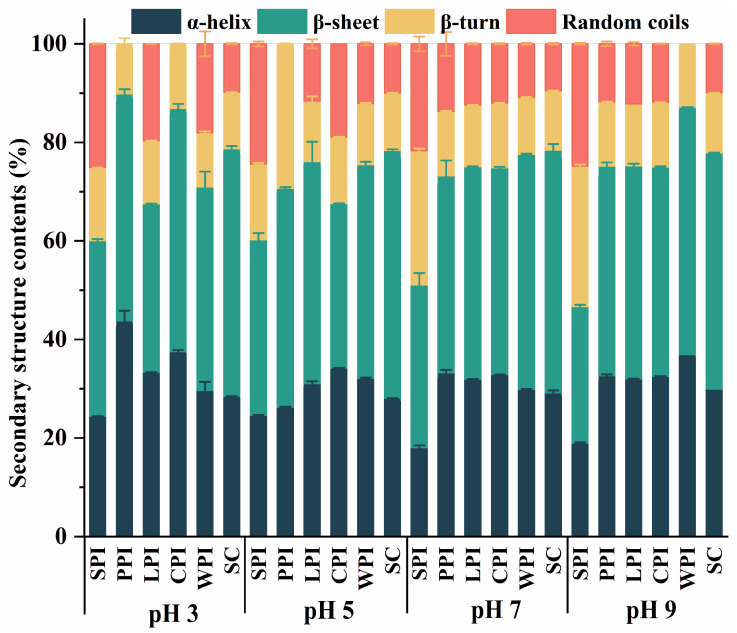
The pH-dependent relative proportion of secondary structures of plant and dairy protein isolates.

Proteins with different functional properties exhibit different structural characteristics [35]. Amide I region was deconvoluted to establish the effect of pH on the secondary structures of plant and dairy proteins, and four major bands were observed: β-sheet (1615–1637 and 1682–1700 cm^−1^), α-helix (1646–1664 cm^−1^), random coil (1637–1645 cm^−1^), and β-turn (1664–1681 cm^−1^) [36]. The major proportion of plant and dairy proteins in Figure 3 was generally represented by β-sheet (27.72–50.29%), followed by α-helix (18.01–43.73%), β-turn (10.14–29.33%), and random coils (0–25.13%), which is consistent with previous reports [37]. It was noted that α-helix and β-sheet of plant proteins exposed to pH 3.0, 5.0, 7.0, and 9.0 generally decreased, whereas β-turn and random coils almost increased at pH 9.0 compared to pH 3.0, demonstrating the weakened rigid structure and increased flexible structures. For instance, when pH adjusted from pH 3.0 to 9.0, the α-helix, β-sheet of SPI dropped by 5.43 and 7.98%, respectively, while the β-turn and random coils of that increased by 13.7 and 0.3%, respectively. In contrast, WPI showed an increase in the α-helix, β-sheet content (7.32 and 8.94%) and decrease in β-turn and random coils by 1.78 and 18.04%, respectively.

### 3.3. Surface Characteristics

#### 3.3.1. Surface Charge

**Figure 4:**
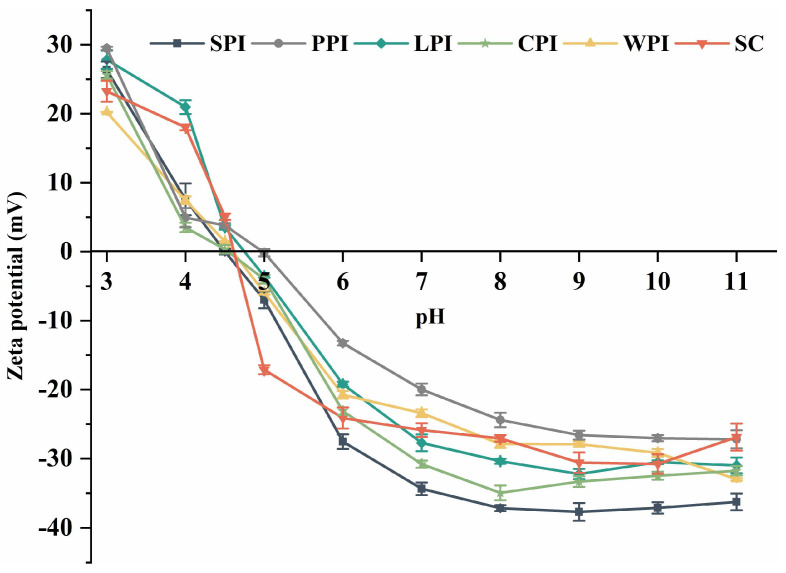
The pH−dependent zeta potential of plant and dairy protein isolates.

The surface charge (ζ-potential) of plant and dairy proteins at different pHs are shown in Figure 4. Surface charge reflects compositional and structural variations of proteins and has an impact on the solubility and emulsifying properties [38]. The surface charge can be affected by several factors, including pH, chemical composition, temperature, and charged compound concentration. Such parameters are influenced by ionic groups (e.g., -COO- and -NH3+), hydrophilic polar groups (e.g., -NH2 and -OH), and non-polar hydrophobic residues (e.g., alkyl and aromatic groups) [39]. The ζ-potential for all protein isolates exhibited a similar pattern, where it was initially positive at pH 3.0 and gradually declined to zero if adjusting pH to their isoelectric point (pI, pH 4–5), and then showed a negative trend (pH 5–11) with further increasing pH level. This is due to amino acid ionization affecting the distribution of charged groups by pH, thus making the carboxyl groups protonated at acidic conditions, and amino groups deprotonated at basic conditions [40].

#### 3.3.2. Effect of pH on Surface Hydrophobicity (Ho)

**Figure 5:**
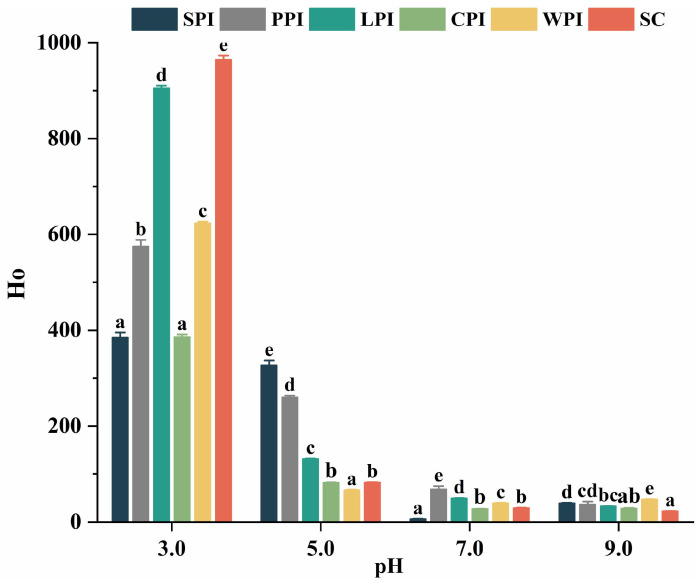
The pH−dependent Ho of legume and dairy protein isolates. Different letters indicated significant difference (*p* < 0.05) among protein samples under different pH treatments.

Surface hydrophobicity (Ho) is used to determine the number of hydrophobic groups on the surface of proteins. This parameter is linked with different functional properties [30,41]. As shown in Figure 5, Ho values were higher at pH 3.0 compared to pH 5.0, and the lowest values were found at pH 7.0 or 9.0. Our results were in agreement with the results that used ANS to measure the Ho of WPI [42] and β-lg [43] at similar pH values. The higher Ho obtained at low pH might be due to more exposure of hydrophobic residues (e.g., aromatic and aliphatic amino acid residues) resulting from the dissociation of protein subunits [39,44]. The anionic probe of ANS, which might interact with positively charged sites of the protein at low pH, could also cause overestimation of Ho at pH 3.0 due to the influence of electrostatic interactions on anionic probe–protein binding [42]. Energy and quantum yield of ANS are primarily affected by molecular rigidity rather than solvent polarity [45]. Ho values of LPI, WPI, and SC were higher than those of other proteins, suggesting that these proteins have unfolded and flexible molecular structures, exposing more hydrophobic sites accessible to ANS. Protein isolates with higher surface hydrophobicity possess better surfactant properties, however the denaturation of the globular proteins may also expose hydrophobic regions [46,47].

#### 3.3.3. Intrinsic Fluorescence Analysis

**Figure 6:**
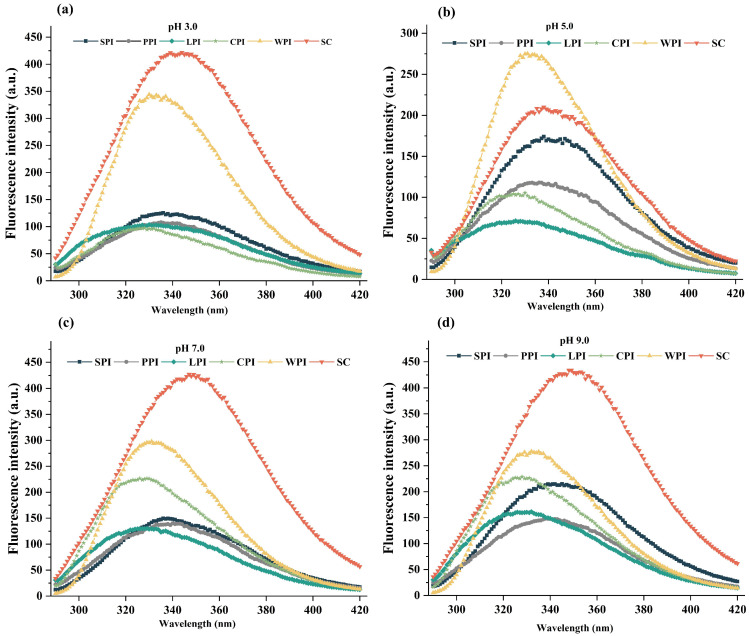
The pH−dependent FI (**a**–**d**) of legume and dairy protein isolates.

Intrinsic fluorescence was measured for determining the tertiary structure of proteins due to the predominant fluorescence in the emission region of tryptophans (Trp) found in proteins, allowing them to reflect the polarity changes of the environment [48,49]. Fluorescence intensity (FI) and maximum emission wavelength (λ_max_) of plant and dairy proteins at different pH values (3.0, 5.0, 7.0, 9.0) are depicted in Figure 6a–d. In general, a Trp is assumed to be buried in a nonpolar environment if its fluorescence maximum is less than 330 nm and considered to be in polar environments if it is higher than 330 nm [50,51]. The results indicated that the effect of pH on intrinsic fluorescence depends on protein sources [39]. Moreover, the λ_max_ of all protein isolates showed a red shift when pH shifted away from 5.0 (near pI), especially for SC, which shifted in the sequence of 338 nm (pH 5.0), 344 nm (pH 3.0), 347 nm (pH 7.0), 349 nm (pH 9.0). Red shifting was reported during acidic and alkaline treatments in comparison to those exposed to natural and near their pI [19]. This phenomenon suggested that the microenvironment where the Trp residues are located has evolved from being hydrophobic to being more polar and hydrophilic [52,53], which usually has a negative impact on the fluorescence intensity (FI) of Trp groups.

However, almost all protein isolates reached maximum FI at alkaline conditions compared with pH near to their pI, which has also been confirmed by previous studies [50]. It might be due to the formation of aggregates at pH 5 that bury most intrinsic tryptophan residues, while electrostatic repulsion results in their migration to polar surfaces at alkaline pH environment [19]. Dairy proteins, especially SC, were higher in FI at tested pH levels (pH 3.0, 7.0, 9.0) than plant proteins. This is possibly due to the rheomorphic and poorly defined tertiary structure of SC that exposes more tryptophan residues compared to plant proteins, which contain more intrinsic tryptophan residues hidden in their globular structures [54]. However, superior interface properties of proteins might be rendered by their greater exposure of hydrophobic groups [50,55]. Therefore, the observed FI might be the result of a combination of these factors.

### 3.4. Techno-Functional Properties

#### 3.4.1. Effect of pH on Protein Solubility

**Figure 7:**
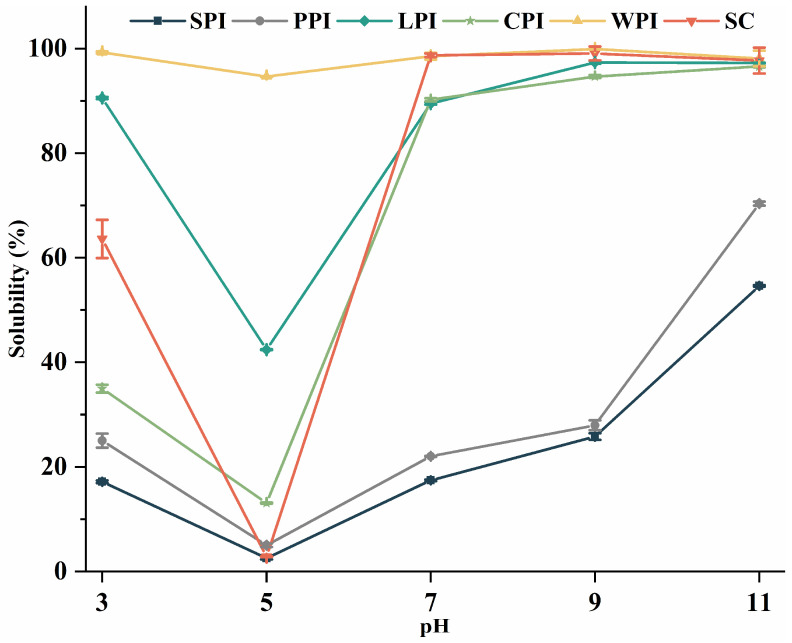
The pH−dependent solubility of plant and dairy protein isolates.

Protein solubility, which is determined by its hydrophilic–lipophilic balance and thermodynamic interaction with water, is a crucial factor for functional properties (e.g., emulsification, foaming, and gelation) of proteins due its ability to hydrate and solubilize in water [25]. In addition to protein content and structure, pH also has an influence on the solubility of proteins. The solubility of plant and dairy proteins at different pH values are shown in Figure 7. There was minimal solubility at pH 5.0 (near pI) in all samples with a U-shaped solubility profile. Similar pH-dependent profiles have been described in various proteins, such as hemp [56], mung bean [57], and quinoa [58]. Near to pI, proteins are less charged, leading to weaker repulsive interactions and protein–protein interaction, resulting in the agglutination of proteins and reduced solubility [59]. As pH moves away from pI, proteins possess a greater number of negative or positive net charges, resulting in stronger electrostatic repulsion, preventing protein aggregation and enhancing their solubility [57,60]. Consequently, plant proteins (especially SPI and PPI) became more soluble when pH levels were adjusted from 7 to 9 and 11, while dairy proteins were highly soluble (>90%) at neutral or alkaline pH levels.

As with zeta-potential profiles (Figure 4), the pH profiles are well correlated, suggesting that protein solubility is closely related to the surface charge of protein isolates [40]. Among all proteins, WPI showed the highest solubility over all pH conditions, which was in accordance with previous reports [60], and making it a good candidate for food industry application, especially in acidic foods (e.g., high-protein milk-based beverages). This may be due to methods used for extracting and preparing protein. The solubility of WPC prepared by ultrafiltration and electrodialysis was reported to not be affected by bulk solution pH [61].

Due to the wide range of pI for individual caseins (pH 3.8–5.8), SC showed the lowest solubility at pH 3.0 and 5.0, leading to a precipitate with varying casein compositions and thus soluble casein fractions that differed as well [62]. After pH adjustment to 7 and 9, the solubility of SC increased remarkably due to the negative charge of casein micelles, which results in redevelopment of electrostatic repulsion between them, leading to its redispersion [63]. LPI showed a better solubility than CPI, PPI, while SPI solubility was the lowest. Previous studies have similarly confirmed that lentil protein has a higher solubility [64], and SPI has a lower solubility, especially at lower pH levels [65]. Overall, LPI showed comparable solubility to SPI and PPI, indicating that those isolates have the potential to be applied as alterative proteins to SPI and PPI. However, the low solubility of all these proteins (except WPI) in low-pH conditions makes their application in acidic food products (e.g., protein-fortified and -carbonated beverages) limited.

#### 3.4.2. Effect of pH on Emulsifying Properties

**Figure 8:**
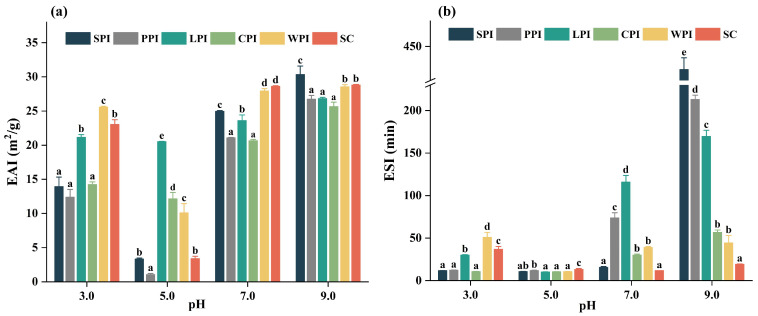
The pH−dependent EAI (**a**) and ESI (**b**) of plant and dairy protein isolates. Different letters indicated significant difference (*p* < 0.05) among protein samples under different pH treatments.

EAI refers to the ability of protein to form emulsions, while the ESI indicates the ability of emulsions to remain in dispersion form and resist separation over a specific period [66]. The results of EAI and ESI of plant and dairy proteins as a function of pH are shown in Figure 8a,b. Overall, all protein isolates generally showed comparable or even superior EAI values at pH 9.0 and 7.0 compared with those at pH 3.0, and lowest at pH 5.0 (near pI). These findings may be determined by similar surface charge (Figure 4) and solubility (Figure 7), and also correspond to previous findings [67]. The lowest EAI at pH 5.0 for all protein isolates are likely due to the lowest solubility near their isoelectric point, causing a less rapid movement of protein to the oil/water interfaces [68]. A possible reason for the enhanced EAI could be attributed to the increased solubility and surface charge of all isolates at neutral and alkaline conditions [20]. Moreover, alkaline conditions have more negative charges, changing electrostatic interactions, and resulting in protein molecules being evenly dispersed in the oil–water emulsion [69].

The EAI of dairy proteins was higher than plant proteins at pH 3.0 and pH 7.0, while that of SPI increased at pH 9.0, possibly due to higher surface charge of SPI (−37.13 mv) than WPI (−27.93 mv) and SC (−34.70 mv). In addition to protein solubility and surface charge, protein composition, conformation state, Ho, and molecular flexibility can also affect the emulsifying properties of proteins [20]. The improved EAI might also be related to the increased content of β-turn and random coils of SPI at pH 9.0. At pH 3.0, LPI showed superior EAI and ESI to other plant proteins, possibly due to its higher solubility and Ho.

At neutral and alkaline conditions, SC showed similar EAI to WPI, which is similar to the results reported previously [70]. ESI of all protein showed similar pH dependence as EAI, with significantly higher ESI at pH 9.0 or 7.0 than 3.0 and 5.0. Furthermore, protein from plant sources had a significantly higher ESI than protein from dairy sources at pH 9.0. Overall, dairy protein showed superior EAI and ESI than that of plant protein at acidic conditions, while plant protein showed comparable EAI and even superior ESI to that of dairy proteins at alkaline conditions.

#### 3.4.3. Effect of pH Foaming Properties

**Figure 9:**
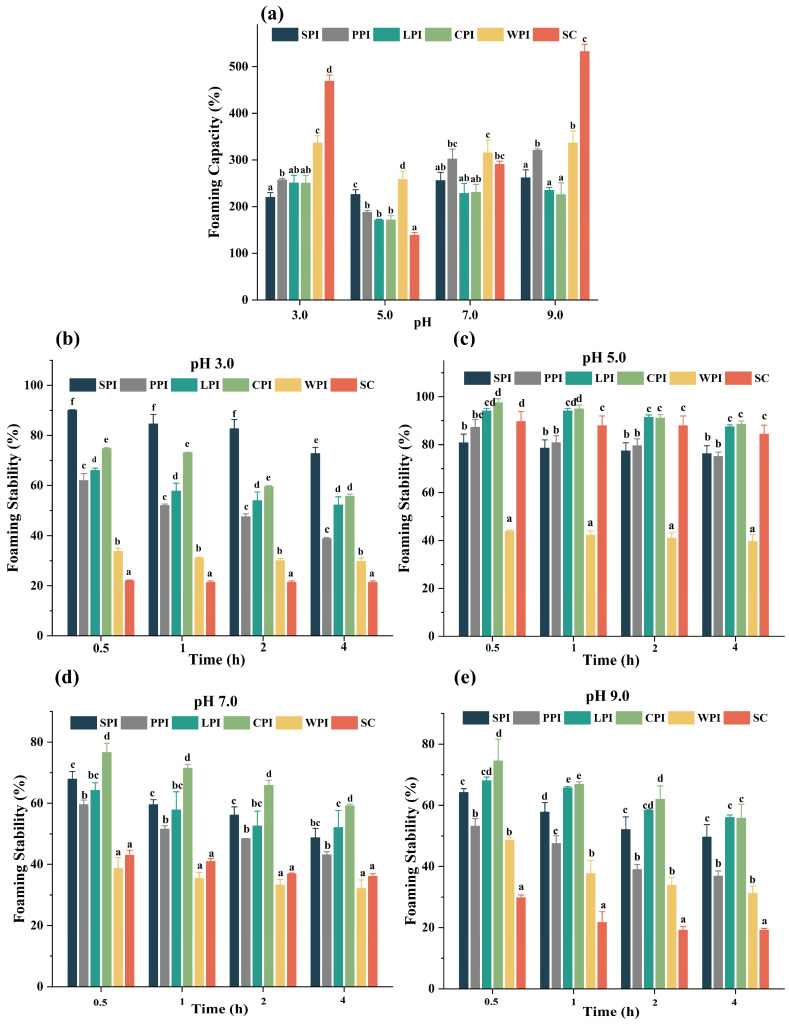
The pH−dependent FC (**a**) and FS (**b**–**e**) of plant and dairy protein isolates. Different letters indicate significant difference (*p* < 0.05) among protein samples under different pH treatments.

Protein foaming properties, which are influenced by solubility and molecular flexibility, are key indicators in the production of meringue, cakes, and ice cream [71,72]. The foaming capacity (FC) of proteins is measured as an increase in interfacial area created during foaming [68]. The FC of plant and dairy proteins at different pH levels are presented in Figure 9a. It can be seen that dairy proteins exhibited higher FC than that of plant proteins at all given pH levels except SC (at pH 5.0), which was the least (138.93 ± 5.56%). Dairy proteins exhibit significantly higher FC (*p* < 0.05), especially at pH 3.0, likely due to their lower molecular weight and higher Ho, therefore encapsulating more air particles due to their rapid diffusion on the air–water surface. Due to its rheomorphic and less-defined tertiary structures (especially β-casein), SC demonstrated considerably (*p* < 0.05) higher FC than WPI at pH 3.0 and 9.0. This makes SC a useful surface-active agent. Based on these results, it can be concluded that lower FC may be associated with low solubility, which is consistent with previous research [59]. FC for all plant proteins were similar in magnitude ranging between 219.88 and 320.45%, whereas FC at pH 5.0 was lower (171.50–226.19%), demonstrating similar FCs and less surface activity across all plant proteins [73]. Overall, FC decreased in the following order: pH 3.0 > pH 9.0 > pH 7.0 > pH 5.0. It is possible that by increasing the net charge of proteins, the hydrophobic interactions are weakened and proteins become more flexible, leading to foam formation [59].

Foam stability (FS) refers to the ability to hold air bubbles under gravity, which is affected by protein properties, viscosity, cohesiveness, pH, and temperature [74]. The FS of proteins is related to the properties of their membrane around the bubble, and bubbles with adhesive, elastic, and airtight membranes can facilitate the improvement of FS for proteins. Figure 9b–e showed that almost all proteins did not exhibit good FC, but they exhibited a relatively high FS compared to other pH levels near the isoelectric point (pH 5.0). This can be explained by electrostatic attraction between molecules that increases the thickness and rigidity of protein films adsorbed on the air–water interface. The results were similar to the previous report [59], which also recorded the lowest FC and highest FS of microalgal proteins at pH 5.0. Plant proteins showed higher FS than dairy proteins at pH away from their isoelectric point. This could be due to the higher molecular weight of globulins in plant proteins, which helps to form adsorption films with good rheological properties and stable foams. SC showed lower FS than WPI at acidic and alkaline conditions. This could be attributed to the curled and soft structure of β-casein in SC which can reduce surface tension and interfacial tension, resulting in foam formation. However, the protein-adsorbed membrane is too thin for foam stability. Therefore, proteins with good FS and FC should have a suitable balance between their soft and rigid structures.

#### 3.4.4. Effect of pH on Water-Holding Capacity (WHC) and Oil-Holding Capacity (OHC)

**Figure 10:**
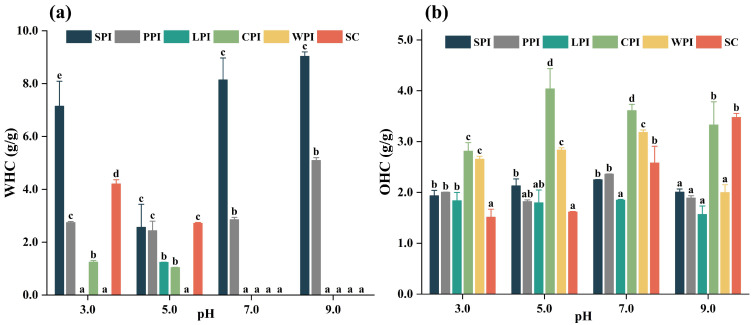
The pH−dependent WHC (**a**) and OHC (**b**) of plant and dairy protein isolates. Different letters indicated significant difference (*p* < 0.05) among protein samples under different pH treatments.

The water-holding capacity (WHC) of a protein measures its ability to retain water under external forces. WHC of all protein isolates, as a function of pH, are shown in Figure 10a. WHC was not evaluated for some samples (especially WPI and LPI) as they were completely soluble in water and showed no water retention capability, showing their limitation in structuring some foods (e.g., meat replacers). These findings were consistent with the results in Sect. 3.4.1. There was a gradual increase in WHC for the evaluated samples when pH was far from their isoelectric points. This phenomenon agrees with another report [25], and likely resulted from a change in protein conformation that exposes more water-binding sites, increasing protein polarity, electric charge, and the proportion of proteins bound in water [68]. It was noted that SPI displayed significantly superior WHC to other protein isolates, followed by PPI at neutral and alkaline conditions. SC also displayed good WHC at acidic conditions, suggesting their suitability for viscous or bakery foods.

Oil-holding capacity (OHC) indicates the ability to absorb or retain oil, and it can help to improve mouthfeel and flavor retention for various food applications [59]. As shown in Figure 10b, the highest OHC was observed for CPI (2.81–4.04 g oil/g protein), followed by SC (1.51–3.48 g oil/g protein) and WPI (2.00–3.18 g oil/g protein). It is believed that OHC is caused by binding of nonpolar side groups of proteins, leading to oil entrapment. In fact, OHC is therefore influenced by the amount of hydrophobic amino acids that are exposed as well as the amount of hydrophobic amino acids in the protein [25]. With the exception of CPI, no significant difference was found between SPI, PPI, and LPI for their OHC at pH 3.0, 5.0, and 9.0, and LPI had a lower OHC at pH 7.0 compared with other samples. Overall, CPI might be used to substitute dairy proteins in many food applications (e.g., meat replacement, meat extenders, and sausages).

### 3.5. Hierarchical Cluster Analysis

**Figure 11:**
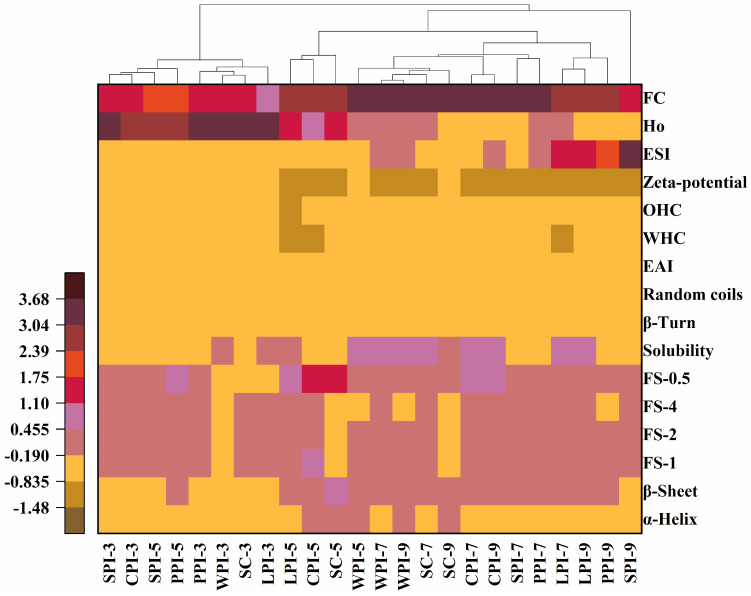
Two−dimensional hierarchical cluster analysis of structural and functional properties from plant and dairy protein at different pH conditions. Sample name with number represents the protein isolates under the corresponding pH treatment.

A two-dimensional hierarchical cluster analysis (HCA) was used to investigate the discrimination of pH dependency among plant and dairy proteins based on their secondary structure, surface properties (zeta potential, Ho), as well as their functional properties (solubility, emulsion, FC/FS, WHC/OHC). Columns with plant and dairy protein isolates with different pH treatments were clustered simultaneously with row data normalized to Z-scores, which determined their color differences. As shown in Figure 11, plant and dairy proteins at different pH conditions were sorted into two main clusters and four subclusters. The first cluster includes all protein samples at pH 3.0, along with some plant proteins (SPI and PPI) at pH 5.0, and the second cluster contains the rest of proteins at pH 5.0, 7.0, and 9.0. Interestingly, the second one can be further divided into two subclusters, with subcluster 3 comprising SPI alone at pH 9.0, which highlights the significant difference in functionality of SPI under alkaline treatment. Subcluster 2 can be further classified into two groups: one group consists of proteins (LPI, CPI, and SC) at pH 5.0, and the other group is composed of plant and dairy proteins at pH 7.0 and 9.0. Overall, the heatmap analysis suggested that pH had greater effect on the structural and functional properties of proteins compared to protein sources, particularly at acidic conditions (pH 3.0 and 5.0), which is consistent with the individual results obtained in this study.

## 4. Conclusions

In this study, we have evaluated the physiochemical, structural, and functional properties of plant and dairy proteins under different pHs. The findings of this present study suggested that there is a great variation between the physicochemical, structural, and functional properties of plant and dairy proteins, in particular under different pH conditions. In general, plant proteins had a darker color, lower solubility, emulsifying properties, and FC, but their FS and WHC were higher than those of dairy proteins. The results of hierarchical cluster analysis indicate that protein function is more affected by pH than source of protein (especially under acidic conditions). Among plant proteins, LPI showed good emulsifying properties at acidic pH levels, while SPI performed better at neutral and alkaline pH levels. SPI and CPI might be suitable for breads, cakes, muffins, and sausages due to their good WHC and OHC, respectively. In addition, the good FC and FS of PPI and CPI, respectively, might make them suitable candidates for some applications, such as ice cream, cakes, and meringue. In conclusion, this study found that various plant proteins have different functional properties and might help select the right source of protein to benefit the design of desirable dairy alternatives products. It is suggested to conduct further research on how to enhance their functional characteristics and expand their commercial applications to real food systems.

## Figures and Tables

**Table 1:** Proximate composition of legume and dairy protein isolates.

Samples	Composition (%)				
	Protein	Moisture	Ash	Lipid	Carbohydrates
SPI	89.96 ^c^ ± 0.26	4.27 ^b^ ± 0.02	4.74 ^c^ ± 0.36	0.94 ^b^ ± 0.02	0.09 ^a^ ± 0.08
PPI	78.66 ^a^ ± 0.04	7.59 ^e^ ± 0.01	4.30 ^bc^ ± 0.03	3.64 ^c^ ± 0.09	5.88 ^d^ ±0.01
LPI	89.07 ^b^ ± 0.79	1.48 ^a^ ± 0.14	4.22 ^b^ ± 0.02	0.67 ^ab^ ± 0.03	4.54 ^c^ ± 0.76
CPI	87.16 ^c^ ± 0.64	1.42 ^a^ ± 0.02	4.68 ^c^ ± 0.01	4.61 ^d^ ± 0.42	2.44 ^b^ ± 0.82
WPI	86.93 ^b^ ± 0.40	5.37 ^c^ ± 0.05	2.72 ^a^ ± 0.22	0.36 ^a^ ± 0.07	4.58 ^c^ ± 0.25
SC	87.51 ^b^ ± 0.49	6.51 ^d^ ± 0.01	4.14 ^b^ ± 0.02	0.66 ^ab^ ± 0.04	1.16 ^a^ ± 0.52

Different letters indicated significant different (*p* < 0.05) among protein samples under neutral conditions.

## Data Availability

Data are contained within the article.
